# Supplementary material for: Empirical methods for controlling false positives and estimating confidence in ChIP-Seq peaks
Source: BMC Bioinformatics. 2008 Dec 5;9:523. doi: 10.1186/1471-2105-9-523 (PMC2628906; doi:10.1186/1471-2105-9-523)
Supplement: Additional File 1 — A variety of html documents from the USeq web site detailing the available applications, their best usage, output file type descriptions, command line menus, etc. [file 1471-2105-9-523-S1.zip › USeqUserGuides/JavaDoc.zip!/JavaDoc/trans/misc/Util.html]

Util


|  |  |  |
| --- | --- | --- |
| **Overview** \| **Package** \| **Class** \| **Use** \| **Tree** \| **Deprecated** \| **Index** \| **Help** | |  |
| **PREV CLASS**   **NEXT CLASS** | **FRAMES**    **NO FRAMES**     **All Classes** |  |
| SUMMARY: NESTED \| FIELD \| CONSTR \| METHOD | DETAIL: FIELD \| CONSTR \| METHOD |  |


---


## trans.misc Class Util

```
java.lang.Object
  trans.misc.Util
```

---

``` public class Util extends java.lang.Object ```

Static helper methods for all things Affy

---

**Constructor Summary**

`Util()`


| **Method Summary** | |
| --- | --- |
| `static int` | `countNumberOfOligoPositions(int[][] startStops)`             Given an array of int[][start, stop] returns the total number of integers, stop included. |
| `static float[][]` | `createVirtualCel(java.io.File celFile)`             Loads a text version cel file into a zeroed virtual chip. |
| `static float[][]` | `createVirtualCel(java.lang.String[] lines)`             Loads a text version cel file into a zeroed virtual chip. |
| `static float[]` | `fetchTextCelFileIntensities(java.io.File celFile)`             Fetches intensity values from a text version cel file. |
| `static java.util.HashMap` | `getChromosomeNumber()`             Creates a HashMap of "chr1"=Byte(1) through "chr22"=Byte(22) plus chrX=23, chrY=24, and chrMT=25. |
| `static java.lang.String[]` | `getNumberChromosome()`             Creates a String[] containing [1]"chr1", [2]"chr2" ... |
| `static Sgr[]` | `loadGrFile(java.io.File grFile, java.lang.String chromName)`             Reads in an sgr file. |
| `static Sgr[]` | `loadSgrFile(java.io.File sgrFile)`             Reads in an sgr file. |
| `static void` | `main(java.lang.String[] args)` |
| `static double[]` | `minMaxWindowArray(Window[] win, int scoreIndex)`             Returns the min and max values of a given score index in a Window[]. |
| `static double[]` | `minMaxWindowScores(Window[] win, int scoreIndex)`             Finds min max score |
| `static java.lang.String` | `parseChromosomeName(java.lang.String name)`             Attempts to parse a chromosome name. |
| `static GrGraph` | `readSimpleGrBarFile(java.io.File simpleBarFile)`             This is a complete hack of Gregg Helt's IGB code to parse bar files. |
| `static java.util.HashMap<java.lang.String,java.lang.String>` | `readTagValPairs(java.io.DataInput dis, int pair_count)`             Methodfor parsing tag values from a binary stream. |
| `static Window[]` | `removeLowOligoWindows(Window[] win, int minNumberOligos)`             Removes windows that contain < minNumberOligos. |
| `static void` | `sortIntervalsBySubWindowMedianRatio(Interval[] intervals)`             Sorts an array of Interval by the median ratio of the best SubWindow. |
| `static java.util.HashMap` | `splitIntervalArrayListByChromosome(java.util.ArrayList intervals)`             Splits an ArrayList containing Interval by chromosome into a HashMap. |
| `static Window[][]` | `splitWindowsByChromosome(Window[] win)` |
| `static boolean` | `writeSimpleBarFile(GrGraph gr, java.io.File barFile)`             Writes a simple bar format graph for a xxx.gr type graph. |
| `static boolean` | `writeSimpleBarFile(java.lang.String chromosome, java.lang.String versionedGenome, java.lang.String strand, int[] basePositions, float[] intensityValues, java.io.File barFile)`             Writes a simple bar format graph for a xxx.gr type graph. |

**Methods inherited from class java.lang.Object**

`equals, getClass, hashCode, notify, notifyAll, toString, wait, wait, wait`

**Constructor Detail**

### Util

```
public Util()
```


**Method Detail**

### main

```
public static void main(java.lang.String[] args)
```

---


### sortIntervalsBySubWindowMedianRatio

```
public static void sortIntervalsBySubWindowMedianRatio(Interval[] intervals)
```

:   Sorts an array of Interval by the median ratio of the best SubWindow. If a sub is not
    found then the score is set to best window score index 1.
    Modifies the original array.
    Sets the sortBy field to the median ratio score.

---


### splitIntervalArrayListByChromosome

```
public static java.util.HashMap splitIntervalArrayListByChromosome(java.util.ArrayList intervals)
```

:   Splits an ArrayList containing Interval by chromosome into a HashMap.

---


### minMaxWindowScores

```
public static double[] minMaxWindowScores(Window[] win,
                                          int scoreIndex)
```

:   Finds min max score

---


### readSimpleGrBarFile

```
public static GrGraph readSimpleGrBarFile(java.io.File simpleBarFile)
```

:   This is a complete hack of Gregg Helt's IGB code to parse bar files. It only works on single graph xxx.gr type files,
    ie two columns, ints and floats.
    Is compatable with TiMAT's Gr2Bar writer.

---


### readTagValPairs

```
public static java.util.HashMap<java.lang.String,java.lang.String> readTagValPairs(java.io.DataInput dis,
                                                                                   int pair_count)
                                                                            throws java.io.IOException
```

:   Methodfor parsing tag values from a binary stream.

    :   **Throws:**: `java.io.IOException`

---


### writeSimpleBarFile

```
public static boolean writeSimpleBarFile(GrGraph gr,
                                         java.io.File barFile)
```

:   Writes a simple bar format graph for a xxx.gr type graph. Modified from IGB code.

---


### writeSimpleBarFile

```
public static boolean writeSimpleBarFile(java.lang.String chromosome,
                                         java.lang.String versionedGenome,
                                         java.lang.String strand,
                                         int[] basePositions,
                                         float[] intensityValues,
                                         java.io.File barFile)
```

:   Writes a simple bar format graph for a xxx.gr type graph. Modified from IGB code.

    :   **Parameters:**: `chromosome` - - ie 'chr21': `versionedGenome` - - ie 'hg18' or 'H\_sapiens\_Mar\_2006' where possible follow UCSC: `strand` - - '+, -, or .' no exceptions. This is written out as a sequence tag value pair.

---


### parseChromosomeName

```
public static java.lang.String parseChromosomeName(java.lang.String name)
```

:   Attempts to parse a chromosome name. Returns null if it fails.
    Looks for chr1-22, chrX, chrY, chrM, and chrMT.

---


### getChromosomeNumber

```
public static java.util.HashMap getChromosomeNumber()
```

:   Creates a HashMap of "chr1"=Byte(1) through "chr22"=Byte(22) plus
    chrX=23, chrY=24, and chrMT=25.

---


### getNumberChromosome

```
public static java.lang.String[] getNumberChromosome()
```

:   Creates a String[] containing [1]"chr1", [2]"chr2" ... [23]"chrX",
    [24]"chrY", [25]"chrM", [26]"chrM", [27]"chrMT", and [28]"chrCtrls".
    [0] is empty.

---


### minMaxWindowArray

```
public static double[] minMaxWindowArray(Window[] win,
                                         int scoreIndex)
```

:   Returns the min and max values of a given score index in a Window[].

    :   **Returns:**: double[]{min, max}

---


### removeLowOligoWindows

```
public static Window[] removeLowOligoWindows(Window[] win,
                                             int minNumberOligos)
```

:   Removes windows that contain < minNumberOligos.

---


### loadSgrFile

```
public static Sgr[] loadSgrFile(java.io.File sgrFile)
```

:   Reads in an sgr file.

---


### loadGrFile

```
public static Sgr[] loadGrFile(java.io.File grFile,
                               java.lang.String chromName)
```

:   Reads in an sgr file.

---


### fetchTextCelFileIntensities

```
public static float[] fetchTextCelFileIntensities(java.io.File celFile)
```

:   Fetches intensity values from a text version cel file.

---


### createVirtualCel

```
public static float[][] createVirtualCel(java.io.File celFile)
```

:   Loads a text version cel file into a zeroed virtual chip.

---


### createVirtualCel

```
public static float[][] createVirtualCel(java.lang.String[] lines)
```

:   Loads a text version cel file into a zeroed virtual chip.

---


### countNumberOfOligoPositions

```
public static int countNumberOfOligoPositions(int[][] startStops)
```

:   Given an array of int[][start, stop] returns the total number of integers, stop included.

---


### splitWindowsByChromosome

```
public static Window[][] splitWindowsByChromosome(Window[] win)
```


---


|  |  |  |
| --- | --- | --- |
| **Overview** \| **Package** \| **Class** \| **Use** \| **Tree** \| **Deprecated** \| **Index** \| **Help** | |  |
| **PREV CLASS**   **NEXT CLASS** | **FRAMES**    **NO FRAMES**     **All Classes** |  |
| SUMMARY: NESTED \| FIELD \| CONSTR \| METHOD | DETAIL: FIELD \| CONSTR \| METHOD |  |


---
